# Massive Spontaneous Pneumothorax

**DOI:** 10.7759/cureus.20992

**Published:** 2022-01-06

**Authors:** Trilok Stead, Joyce Lee, Derrick Huang, Jesse DeLosSantos, Latha Ganti

**Affiliations:** 1 Emergency Medicine, Trinity Preparatory School, Winter Park, USA; 2 Emergency Medicine, Brown University, Providence, USA; 3 Emergency Medicine, Ocala Regional Medical Center, Ocala, USA; 4 Emergency Medicine, Lakeland Regional Health Medical Center, Lakeland, USA; 5 Emergency Medicine, Envision Physician Services, Plantation, USA; 6 Emergency Medicine, University of Central Florida College of Medicine, Orlando, USA; 7 Emergency Medicine, HCA Healthcare Graduate Medical Education Consortium Emergency Medicine Residency Program of Greater Orlando, Orlando, USA

**Keywords:** chest radiography, chest tube, pneumothorax, emergency medicine, primary spontaneous pneumothorax

## Abstract

Spontaneous pneumothorax (SP) is an abnormal occurrence in which air enters the pleural space, increasing pressure that pushes on the lung, causing it to collapse in part or full. Diagnosis is made by physical exam and can be confirmed by chest X-ray (CXR), chest computed tomography (CT), ultrasonography, and other forms of imaging showing a collapsed lung. We present the case of a 35-year-old male cannabis user presenting with sharp sudden pains in the right chest, who presented almost 12 hours after symptom onset and was diagnosed with a 90% pneumothorax of the right lung. Symptoms, diagnosis, treatment, and risk factors are discussed.

## Introduction

Pneumothorax is defined as the presence of air in the pleural space, a thoracic structure delineated by the visceral and parietal pleura in between the lung and chest wall [1]. Under normal conditions, there is no passage for air to enter this space, and thus the pressure in the pleural space remains very low, with higher pressures in the airway keeping the lungs inflated. When this isolation becomes compromised, the lungs become compressed from the external air pressure, causing them to collapse. Air can enter the pleural space in three ways: alveolar release, communication between the pleura and the environment, or the presence of gas-producing organisms in the cavity [2]. A case of pneumothorax in which there are no obvious precipitating factors present is referred to as spontaneous or atraumatic pneumothorax. 

There are two types of SP: the first is known as primary spontaneous pneumothorax (PSP), which is defined as SP without any underlying condition. The second is secondary spontaneous pneumothorax (SSP), which is SP that occurs due to an underlying pulmonary condition. Common causes of SSP include tuberculosis and chronic obstructive pulmonary disease (COPD) [3]. Populations at risk for PSP include young, thin, male patients, smokers (especially cannabis smokers). SP often occurs in individuals at rest, not during vigorous exercise [4]. Symptoms of PSP include sudden ipsilateral chest pain and dyspnea. Imaging can detect the presence of air in the pleural cavity. Treatment for SP varies depending on the magnitude of the case. For more benign cases of pneumothorax, the intervening air is removed through simple aspiration, while in more serious cases, interventions such as valve insertion, chest tube placement, or thoracotomy are considered [5].

## Case presentation

The patient is a 35-year-old male who noticed discomfort in the right chest the night before post-eating. The chest pain was not associated with shortness of breath, fever, or chills. He stated that while taking a deep breath, it made him feel like he had to cough. The pain did not worsen upon twisting or palpation. The patient endorsed smoking cigarettes and marijuana but denied cocaine or other drug use. The patient denied nausea, vomiting, abdominal pain, urinary symptoms, or headache. The patient had no known COVID-19 exposure. 

**Figure 1:**
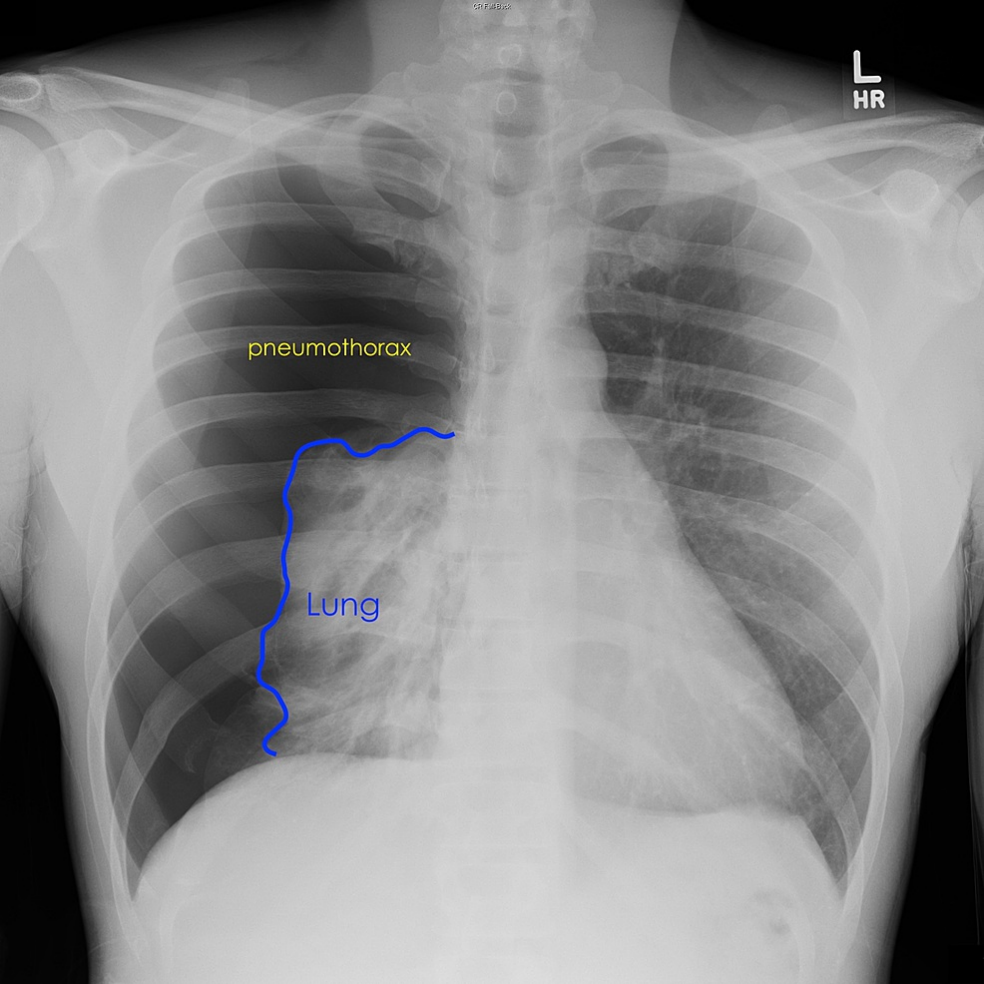
Chest radiograph demonstrating a 90% right-sided pneumothorax

Physical examination of the apprehensive patient revealed absent right lung sounds and shallow breathing. Laboratory analysis was unremarkable. Chest radiograph showed a near-complete lung collapse [Figure 1].

The patient’s vital signs were pulse oximetry 99% on room air, blood pressure 125/74 mmHg, pulse 68 beats per minute, respiratory 17 breaths per minute, and temperature 37.2˚C. 

**Figure 2:**
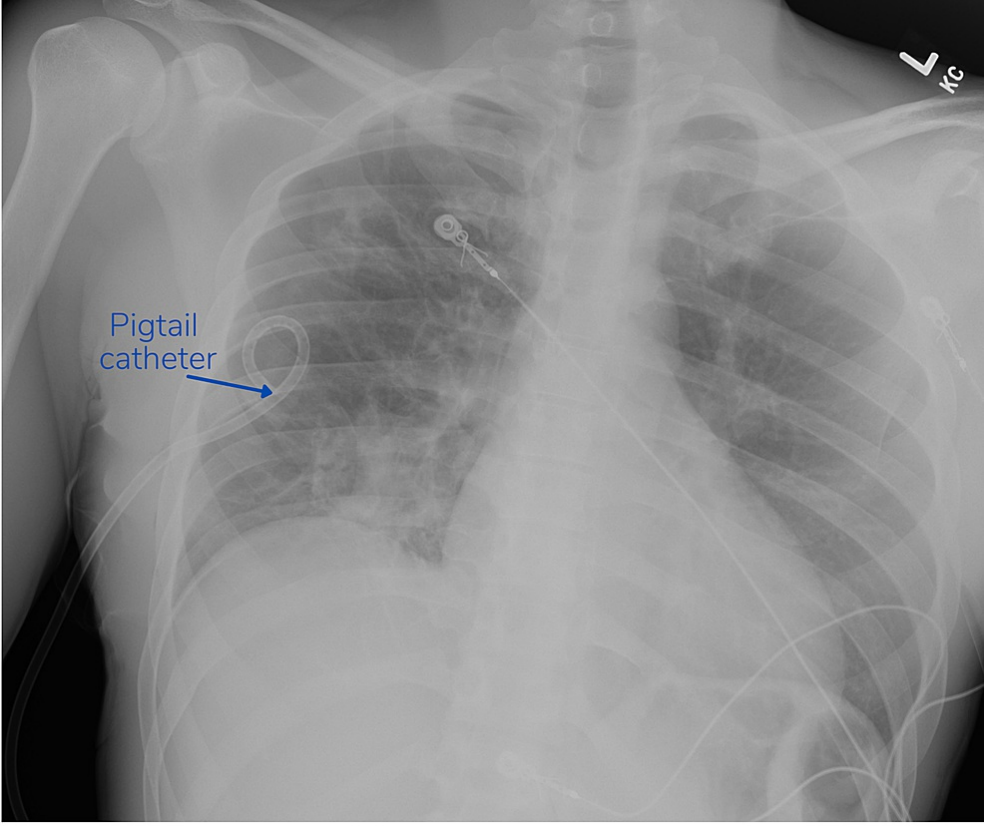
Chest radiograph taken two hours and twenty-five minutes after initial presentation demonstrating resolution of pneumothorax following pigtail catheter placement

A pigtail catheter was promptly placed using local anesthesia. The patient’s breathing improved significantly. A follow-up chest radiograph two hours and twenty-five minutes later demonstrated resolution of the pneumothorax [Figure 2]. 

**Figure 3:**
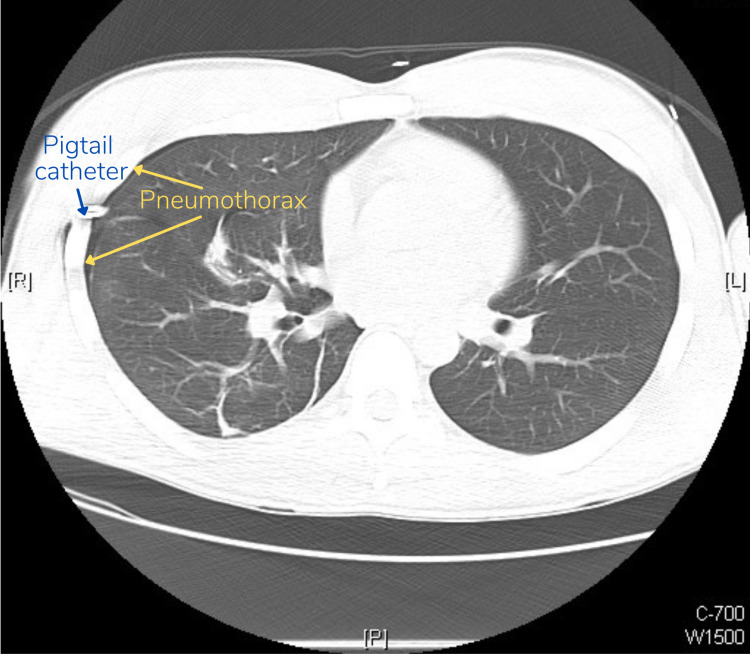
Chest CT performed three hours after initial presentation demonstrating small, residual pneumothorax (5%) following pigtail catheter placement

A follow-up chest CT conducted several minutes later to evaluate the lung parenchyma revealed that there was still a small (5%) residual pneumothorax in addition to blebs-small sacs of air from ruptures in the lung tissue, which likely were the cause for the patient’s SP [Figure 3].

The patient was admitted overnight for observation with supplemental oxygen, as he was highly anxious about possible pain associated with the chest catheter. He was discharged the next day, feeling well. He was counseled about marijuana and tobacco use as risk factors for spontaneous pneumothorax.

## Discussion

Spontaneous pneumothorax is a potentially life-threatening condition that can affect otherwise healthy individuals. This unassuming etiology for SP often results in overlooked risk. Treatment for most general pneumothoraces involves tube or catheter drainage of the air that fills the pleural cavity, causing the lung to collapse. Diagnosis of the condition is straightforward, as it only requires basic imaging tools to visualize a collapsed lung-as seen in this case, a plain CXR makes the diagnosis. 

Our patient showed no signs of an underlying pulmonary condition that could have provided a reason for his SP, such as tuberculosis or chronic obstructive pulmonary disease (COPD), and no direct or indirect communication between the pleural cavity and the atmosphere was observed. However, he did smoke marijuana, and this is a known risk factor. A retrospective study of 67 patients showed that cannabis usage is associated with SP recurrence and the eventual need for operative intervention [6]. Emergent treatment of the patient’s SP through catheter placement was possible due to prompt diagnosis via near absent breath sounds on the affected side and confirmation via chest radiography. 

Although not used in our patient’s case, point-of-care ultrasonography (POCUS) is another imaging modality that can be used to rapidly diagnose or rule out pneumothorax at the bedside [6]. The development of the e-FAST (extended focused assessment with sonography for trauma) protocol allows the use of POCUS to look for free intraperitoneal fluid, free fluid in the pelvis, pericardial fluid, pleural effusion, and notably, pneumothorax [7]. In a study comparing e-FAST to CXR, the e-FAST showed higher sensitivity over CXR in detecting post-traumatic pneumothoraces (48.8% versus 20.9%) [8]. CT, however, has the highest sensitivity for detecting pneumothoraces, especially when they are small, and can also reveal blebs. This is highlighted in our patient's case, where the follow-up chest radiograph demonstrated resolution of the pneumothorax, but the CT demonstrated a small residual one. Leaving pneumothorax untreated could lead to respiratory failure and pneumomediastinum, amongst other life-threatening complications [9].

## Conclusions

Spontaneous pneumothorax is a potentially life-threatening condition that can be seen in healthy individuals. When treating spontaneous pneumothorax, prompt evacuation of the trapped air in the pleural space is imperative. Because spontaneous pneumothorax often occurs in otherwise healthy individuals, it is important to consider risk factors and underlying lung abnormalities and to quickly use a physical exam and prompt imaging to diagnose and treat the condition.
